# ADJUVANT CHEMORADIOTHERAPY AFTER SUBTOTAL OR TOTAL GASTRECTOMY AND D2 LIMPHADENECTOMY INCREASES SURVIVAL IN ADVANCED GASTRIC CANCER?

**DOI:** 10.1590/0102-672020190001e1464

**Published:** 2019-12-20

**Authors:** Nelson Adami ANDREOLLO, Eric DRIZLIONOKS, Valdir TERCIOTI-JUNIOR, João de Souza COELHO-NETO, José Antonio Possato FERRER, José Barreto Campello CARVALHEIRA, Luiz Roberto LOPES

**Affiliations:** 1 Digestive Diseases Surgical Unit and Gastrocenter, Campinas, SP, Brazil; 2 Division of Oncology, Department of Surgery and Internal Medicine, School of Medical Sciences, State University of Campinas - UNICAMP, Campinas, SP, Brazil

**Keywords:** Gastric neoplasm, Adjuvant therapy, Gastrectomy, Neoplasias gástricas, Terapias adjuvantes, Gastrectomia

## Abstract

**Background:**

The treatment of advanced gastric cancer with curative intent is essentially surgical and chemoradiotherapy is indicated as neo or adjuvant to control the disease and prolong survival.

**Aim:**

To assess the survival of patients undergoing subtotal or total gastrectomy with D2 lymphadenectomy followed by adjuvant chemoradiotherapy.

**Methods:**

Were retrospectively analyzed 87 gastrectomized patients with advanced gastric adenocarcinoma, considered stages IB to IIIC and submitted to adjuvant chemoradiotherapy (protocol INT 0116). Tumors of the esophagogastric junction, with peritoneal implants, distant metastases, and those that had a compromised surgical margin or early death after surgery were excluded. They were separated according to the extention of the gastrectomy and analyzed for tumor site and histopathology, lymph node invasion, staging, morbidity and survival.

**Results:**

The total number of patients who successfully completed the adjuvant treatment was 45 (51.7%). Those who started treatment and discontinued due to toxicity, tumor-related worsening, or loss of follow-up were 10 (11.5%) and reported as incomplete adjuvant. The number of patients who refused or did not start adjuvant treatment was 33 (48.3%). Subtotal gastrectomy was indicated in 60 (68.9%) and total in 27 (31.1%) and this had a shorter survival. The mean resected lymph nodes was 30.8. Staging and number of lymph nodes affected were predictors of worse survival and the more advanced the tumor. Patients undergoing adjuvant therapy with complete chemoradiotherapy showed a longer survival when compared to those who did it incompletely or underwent exclusive surgery. On the other hand, comparing the T4b (IIIB + IIIC) staging patients who had complete adjuvance with those who underwent the exclusive operation or who did not complete the adjuvant, there was a significant difference in survival.

**Conclusion:**

Adjuvant chemoradiotherapy presents survival gain for T4b patients undergoing surgical treatment with curative intent.

## RESUMO

**Racional:**

O tratamento do câncer gástrico avançado com intenção curativa é essencialmente cirúrgico e a quimiorradioterapia está indicada como neo ou adjuvância para controlar a doença e prolongar a sobrevida.

**Objetivos:**

Avaliar a sobrevida dos doentes submetidos à gastrectomia subtotal ou total com linfadenectomia D2 seguidos de quimiorradioterapia adjuvante.

**Métodos:**

Foram analisados retrospectivamente 87 gastrectomizados portadores de adenocarcinoma gástrico avançado considerandos estádios IB até IIIC e submetidos à quimiorradioterapia adjuvante (protocolo INT 0116). Foram excluídos os tumores da transição esofagogástrica, com implantes peritoneais, metástases à distância e os que após a operação apresentaram margem cirúrgica comprometida ou óbito precoce. Foram separados quanto à extensão da gastrectomia e analisados em relação ao local e histopatologia do tumor, invasão linfonodal, estadiamento, morbidade e sobrevida*.*

**Resultados:**

O número de doentes que conseguiu completar o esquema adjuvante na sua totalidade foi de 45 (51,7%). Os que iniciaram o tratamento e interromperam por toxicidade, piora relacionada ao tumor, ou perda de seguimento foram 10 (11,5%) e relacionados como adjuvância incompleta. O número de doentes que recusou ou não iniciou o tratamento adjuvante foi de 33 (48,3%). A gastrectomia subtotal foi indicada em 60 (68,9%) e a total em 27 (31,1%) e esta apresentou menor sobrevida. A média de linfonodos ressecados foi de 30,8. O estadiamento e o número de linfonodos acometidos foram preditores de pior sobrevida e quanto mais avançado foi o tumor. Os pacientes submetidos à terapia adjuvante com quimiorradioterapia completa mostraram sobrevida maior quando comparados àqueles que a fizeram de forma incompleta ou submetidos à operação exclusiva. Por outro lado, comparando-se os doentes estádios T4b (IIIB + IIIC) que tiveram adjuvância completa com os submetidos à operação exclusiva ou que não completaram a adjuvância, houve significativa diferença na sobrevida.

**Conclusão:**

- A quimiorradioterapia adjuvante apresenta ganho de sobrevida para doentes em estádio T4b submetidos ao tratamento cirúrgico com intenção curativa.

## INTRODUCTION

Despite the declining incidence in the rest of the world, gastric cancer appears third in incidence among men and fifth in women in Brazil. Due to the aggressiveness of the disease and the symptoms being nonspecific, most patients already have advanced tumors at the time of diagnosis, providing low survival rates^7^ ^,^ ^13^ ^,^ ^34^.

The most effective curative intention treatment for gastric cancer is surgical^25^. Due to the high tumor recurrence rates and poor prognosis, over the past 30 years there has been a strong efforts from the medical community to develop effective cancer therapies - such as chemotherapy and radiotherapy, neoadjuvants or adjuvants - to improve prognosis and long-term survival. Therefore, the treatment of this tumor should be multidisciplinary and individualized. ^8^ ^,^ ^15^ ^,^ ^19^ ^,^ ^26^ ^,^ ^40^.

Surgical treatment is often the first option, and even when there is no evidence of residual disease during surgery, in most cases there is a need for complementation with some adjuvant therapeutic modality^8^. Histological lymph node invasion, positive peritoneal lavage and local tumor tumors are some of the important prognostic factors for survival^31^.

In 2001, the Intergroup-0116 (INT 0116) clinical trial was published, also known as the MacDonald protocol, which compared two groups of randomized patients, surgical treatment followed by postoperative chemoradiotherapy versus exclusive surgical treatment. The recommended chemotherapy was using 5-fluorouracil, leucovorin and radiotherapy was indicated with the total dose of 4500 cGy^21^. The adjuvant protocol showed favorable results and was quickly incorporated as a treatment for gastric cancer in the United States and other western and eastern countries^17^ ^,^ ^20^ ^,^ ^23^ ^,^ ^30^ ^,^ ^41^. However, only 10% of the study patients underwent D2 lymphadenectomy^21^. Thus, there are few studies evaluating the protocol of MacDonald et al.^21^ in the population of patients undergoing D2 lymphadenectomy, usually employed by some Oncology Services, in the Brazilian population.

The aim of this study was to retrospectively analyze the morbimortality and survival of gastric cancer patients who underwent subtotal or total gastrectomy with D2 lymphadenectomy and who submitted to adjuvant therapy.

## METHODS

Medical records of patients with previous diagnosis of advanced gastric adenocarcinoma (noncardia) submitted to surgical treatment with curative intent were selected, treated and followed at the University Hospital of the State University of Campinas, Campinas, SP, Brazil, from January 2002 to August. 2013 and that used the protocol of MacDonald et al. ^21^, postoperatively.

Adjuvant cancer treatment was started on average 45 days after surgical treatment and consisted of 425 mg/m^2^ 5-fluorouracil associated with leucovorin 20 mg/m^2^ for five days, followed by radiotherapy 180 cGy/day for five days for five weeks. The radiotherapy period was performed between the third and fourth cycle of chemotherapy. The dose of 5-fluorouracil was changed to 400 mg/m^2^ and applied on the first four days and last three days of radiotherapy.

A total of 127 cases were identified, with stage IB to IIIC (TNM 2010)^3^, however were excluded those with compromised resection margin (n=15, 11.8%), distant metastases (n=12, 9.4%), peritoneal implants (n=8, 6.2%) and with death up to 30 days (n=5, - 3.9%). The final number of cases included in the study was 87.

In addition to the analysis of morbidity and mortality, information on epidemiological profile (age and gender), tumor location and histopathology, number of lymph nodes resected and affected, angiolymphatic and perineural invasion, and type of procedure were also obtained.

### Statistical analysis

The frequency tables of the categorical variables were made, with absolute (n) and percentage (%) frequency values, and descriptive statistics of numerical variables. Cox regression analysis was used to study survival factors. Kaplan-Meier curves were constructed for survival analysis. The significance level adopted for the study was 5%. The following computer programs were used for statistical analysis: The SAS System for Windows (Statistical Analysis System), version 9.4. SAS Institute Inc, 2002-2008, Cary, NC, USA^9^.

## RESULTS

The results are summarized in Tables 1 and 2. Depending on the tumor site, subtotal (n=60, 68.9%) or total gastrectomy (n=27, 31.1%) and D2 lymphadenectomy were performed, following the protocol of the Japanese Gastric Cancer Association^18^ ^,^ ^19^. The predominant tumor location was in the antrum (n=41, 47.1%) which favored the preference for subtotal resection. The average number of resected lymph nodes was 30.8 per patient, considered adequate^18^ ^,^ ^19^. All were operated at the same institution by the same team of surgeons.

**TABLE 1:** Gender, age range, procedures performed, tumor location and postoperative complications (n=87)

Gender	n
Male	54 (62.0%)
Female	33 (37.9%)
Age range (years)	n
< 40	7 (8.0%)
41 - 50	14 (16.1%)
51 - 60	17 (19.5%)
61 - 70	29 (33.3%)
71 - 80	18 (20.6%)
> 81	2 (2.2%)
Procedures	n
Subtotal gastrectomy + D2 resection	60 (68.9%)
Total gastrectomy + D2 resection	27 (31.1%)
Tumor location	n
Antrum	45 (51.7%)
Body	21 (24.1%)
Antrum and body	14 (16.1%)
Fundus	4 (4.7%)
Fundus and body	3 (3.4%)
Postoperative complications	n
Bowel obstruction	8 (9.1%)
Fistulas	5 (5.7%)
Thrombosis	3 (3.4%)
Cavity abscess	2 (2.2%)
Wall herniation	2 (2.2%)
Cardiopulmonary	2 (2.2%)
Haemorrhage	1 (1.1%)

**TABLE 2:** Staging, survival, histopathological findings, histopathological types, tumor recurrence, and follow-up related to postoperative treatment (n = 87)

Staging (*)	n	Adjuvancy complete	Adjuvancy incomplete	Surgery only
IB	7 (8.0%)	2	0	5
IIA	6 (6.8%)	4	0	2
IIB	14 (16.0%)	5	3	6
IIIA	17 (19.5%)	8	2	7
IIIB	20 (22.9%)	12	3	5
IIIC	23 (26.4%)	13	2	8
Total	87 (100%)	43 (49.4%)	10 (11.5%)	34 (39.1%)
Overall survival (months)	34.3	38.9	2.4	30.1
Hystopathological				
Angiolymphatic positive	49 (56.3%)	30	8	11
Angiolymphatic negative	38 (43.6%)	13	2	23
Perineural positive	36 (41.3%)	20	3	13
Perineural negative	51 (58.6%)	23	7	21
Both positive	28 (32.1%)	18	2	8
Both negative	30 (34.4%)	11	1	18
Hystological type				
Well differentiated	5 (5.7%)	4	1	0
Moderately differentiated	40 (45.9%)	15	5	20
Poorly differentiated	28 (32.1%)	18	3	7
Undifferentiated	14 (16.1%)	6	1	7
Total	87 (100%)	43	10	34

(*) 7ª Classification TNM^3^

**FIGURE 1:**
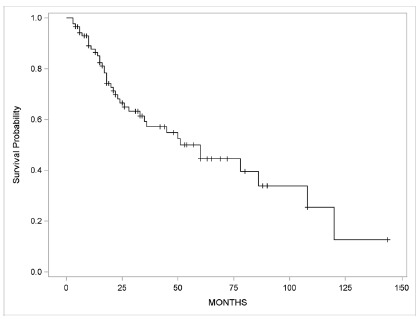
Overall survival

Most patients had tumors at the advanced disease staging - IIIA (n=17, 19.5%), IIIB (n=20, 22.9%) and IIIC (n=23, 26.4%) - totalizing 68.9% of the cases, reflecting the late diagnosis. The most frequent histopathological type was moderately differentiated adenocarcinoma (46%). In addition, 32.1% had both angiolymphatic and perineural invasion (Table 2). Figure 1 shows the overall survival of the 87 patients analyzed.

Average=65.99; median= 60; SD=7.18

**FIGURE 2:**
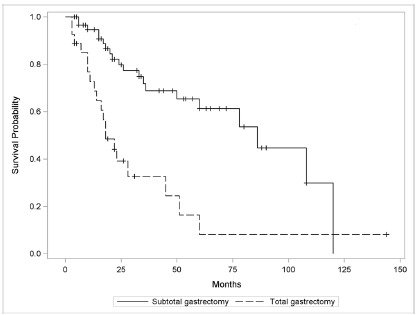
Survival of cases submitted to subtotal gastrectomy (n=60) vs. total gastrectomy (n = 27)

The risk of death comparing total gastrectomy with subtotal gastrectomy was statistically significant (HR=3.36 CI 95%=1.77-6.40 p=0.0002, Figure 2). Worse outcome also occurred for those with advanced staging and greater lymph node involvement. 

Subtotal - average=76.47; median=86; SD7.38. Total - average=27.34; median=18; SD=4.30

The total number of patients who successfully completed the adjuvant protocol was 45 (51.7%). Patients who started the protocol but discontinued due to toxicity, tumor-related worsening, or loss of follow-up were 10 (11.5%), and were considered as incomplete adjuvant. The number of patients who refused or did not start adjuvant treatment was 33 (48.3%).

**FIGURE 3:**
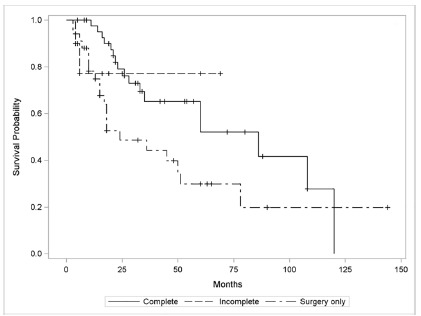
Survival of the three study groups: complete adjuvant treatment (n=43), incomplete adjuvant treatment (n=10) and surgical treatment only (n=34)

**FIGURE 4:**
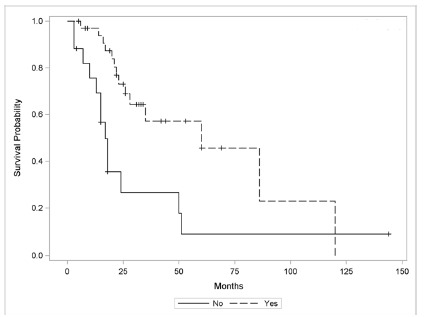
Comparative survival of stage T4b patients (IIIB + IIIC) submitted to adjuvant vs. exclusive operation (p=0.004)

Patients undergoing adjuvant therapy with complete chemoradiotherapy showed longer survival by the Kaplan-Meier curve when compared to those who received incomplete therapy or underwent exclusive surgery; however the result was not statistically significant (HR=2.09, 95% CI=1.09-4.02, p=0.071, Figure 3). On the other hand, when comparing T4b (IIIB + IIIC) patients who had complete adjuvancy with those undergoing T4b exclusive surgery, there was a statistically significant difference in survival (p=0.004, Figure 4). The study of the group that performed incomplete adjuvancy was impaired by the reduced number of patients.

Complete - average=73.26; median=86; SD=8.46. Surgery only - average=38.58; median=24; SD=5.67

Yes - average=63.59; median=60. No - average=31.73; median=17. Overall - average=54.63; median=35

After adjuvant therapy was finished, patients were followed up with frequent outpatient consultations. After two years of surgery the visits were every six months. All patients had clinical follow-up performing abdominal ultrasonography, upper digestive endoscopy, and abdominal, pelvic and chest computed tomography^11^. 

The main site of recurrence was peritoneum (n=12, 13.7%) and liver (n=10, 11.4%). The presence of tumor recurrence was a predictor of worsening patient survival (HR=2.28, 95% CI=1.08-4.81, p=0.029). Comparing staging III with staging IB + IIA, it was found that staging III showed a statistically significantly shorter survival (RR=2.49 95% CI=1.1163-5.339, p=0.018). The number of lymph nodes affected was also a predictor of poor prognosis (HR=1.174, 95% CI=0.465-2.956, p=0.028) in staging III cases. The other comparisons show no statistically significant difference.

## DISCUSSION

The Globocan statistics show that gastric cancer remains important worldwide and was responsible for over 1,000,000 new cases in 2018, with an estimated 73,000 deaths (equivalent to 1 in 12 deaths globally), making it is the fifth most frequently diagnosed and the third leading cause of cancer death. The most common histological type is adenocarcinoma. Among men, it is the most commonly diagnosed tumor and the leading cause of cancer death in many West Asian countries^6^.

Surgical resection followed by adequate lymphadenectomy and detailed analysis of resected lymph nodes remains the best chance of disease control and cure, in early cases. Despite improvements in surgical techniques and more extensive lymphadenectomy, 40% to 60% of patients relapse from 2 to 5 years. Therefore, even when performing R0 surgical resection, there is improvement with the addition of chemotherapy and radiotherapy, necessary and indispensable for increased survival^14^ ^,^ ^31^.

The conversion therapy has recently emerged as an alternative therapy, prolonging survival in patients with advanced neoplastic disease. It consists of oncologic treatments followed by surgery in stage IV patients. It is an option to treat unresectable lesions in patients with distant lymph node metastasis and even with metastatic disease or peritoneal dissemination^32^.

Despite major debates over the past 30 years regarding what would be the best surgical technique and extension of lymphadenectomy, it is currently accepted that resection to D2 yields the best long-term results regarding tumor survival and recurrence. The vast majority of recent large clinical trials perform D2 lymphadenectomy as the basis for curative surgery^22^ ^,^ ^27^ ^,^ ^36^. It is also agreed that the presence of microscopically affected margins is an independent prognostic factor^39^.

The oncologic treatments, neoadjuvant, perioperative or adjuvant, vary considerably and specialists employ various regimens to effectively control the disease. Presently, there is no consensus on which one is most appropriate, considering that the studies performed were heterogeneously designed with different drugs and dose regimens, including chemotherapy alone or in combination with radiotherapy^15^ ^,^ ^19^ ^,^ ^26^ ^,^ ^40^.

The most important studies including neoadjuvant chemotherapy followed by surgery, well controlled and with long follow-up were MAGIC^10^, FFCD 9703^42^ and FLOT-4^1^. Interestingly, most adjuvant chemotherapy treatment studies are in Asian and include ACTS-GC^33^, CLASSIC^24^, SAMIT^38^ and ITACA-S^1^.

To reduce the likelihood of local recurrence of resected gastric cancer, in addition to adjuvant chemotherapy, local treatments employing radiotherapy have been investigated in both neoadjuvant and adjuvant contexts. Thus, the studies that best evaluated this treatment were: INT 0116^21^, ARTIST^28^, POET^37^ and CROSS^16^. 

The INT-0116 study, which established postoperative chemoradiotherapy treatment using 5-fluorouracil, leucovorin and a total dose of 4500 cGy, showed the benefit for patients into the T2N0 staging, which according to the classification included tumors involving the subserosa and muscularis propria, respectively (which would be classified as T3 and T2, according to the current TNM 2010 classification). This study showed a three-year survival rate of 50% in the group receiving chemoradiotherapy vs. 41% in the exclusive surgery treated group (p=0.005) and progression-free survival rate of 48% in the chemoradiotherapy group vs. 31% in the exclusive surgery treated group (p<0.001)^21^. Smalley et al.^35^ in 2012 also demonstrated that the benefits of chemoradiotherapy remained after 10 years of postoperative follow-up and that the results remained favorable in relation to mortality and tumor recurrence.

In this research were selected and included patients from staging T2N0 (IB) and those with any lymph node involvement, in order to make a similar selection to the original article, without esophagogastric junction tumors included. Eighty-seven patients with complete 5-year follow-up were included in the present study, however only 49.1% completed all treatment, 11.5% started treatment and discontinued due to severe side effects and 39.1% did not start the protocol for lack of clinical conditions. Statistical analysis failed to show benefit in terms of survival gain for patients undergoing complete adjuvant treatment considering all stages, although the Kaplan-Meier curve in this group showed a difference in mean survival when comparing patients that performed adjuvant therapy with those who did not (73.26 vs. 38.58 months respectively, Figure 3). It is possible that with a larger sample, the benefit of adjuvant therapy may be statistically significant. On the other hand, when comparing only patiens with T4b staging (IIIB + IIIC) who underwent adjuvant treatment or not, survival gain was demonstrated (Figure 4). Our results also show a statistically significant survival gain in patients undergoing subtotal gastrectomy compared with total gastrectomy (Figure 2). Therefore, as already shown in the literature, there is a significant increase in morbidity when comparing total with subtotal gastrectomy results. Whenever possible, subtotal gastrectomy is preferable, with the surgical margins preserved^4^ ^,^ ^27^.

In addition, patients in staging I and II compared to staging III who did not have recurrence of tumor in the peritoneum and liver when compared to patients who had this type of recurrence, and patients in staging III who did not have lymph nodes involvement compared to patients who had lymph nodes involvement, also had survival gains. The other comparisons did not show statistically significant differences.

The related criticism about the study by MacDonald et al. ^21^ was that only 10% of patients (n=556) underwent a D2 resection^37^. This fact raised doubts regarding the real need for radiotherapy for those undergoing radical resection. Another negative aspect is its toxicity, hematologic toxicity can reach 54% and gastrointestinal toxicity in 33% of patients, with a treatment-related mortality of 1%^17^. 

Similar studies using the same adjuvant protocol reported satisfactory results. Kim et al.^20^ employed this protocol in 544 patients undergoing curative treatment and D2 lymphadenectomy, concluding that 5-year survival rates were consistently longer in stages II, IIIA, IIIB and IV than in the group of 446 patients undergoing exclusive surgery. In addition, the adjuvant treatment employed was associated with increases in median duration of relapse-free survival. Yakir et al.^41^ employed the same protocol in 36 staging T4b patients, concluding that median disease-free survival was 37.4 months and overall survival was 40.3 months, with no deaths related to treatment toxicity. Montenegro et al.^23^ used the same protocol in 84 patients with advanced gastric adenocarcinoma who underwent gastric resection and D2 lymphadenectomy, concluding that the average 3-year survival was 23.9%; however, analyzing the groups for lymph node invasion, they found that in the same period, N1 survival was 100%, N2 was 51.9% and N3 was 16.3%. They conclude that the adjuvant protocol used reduced the risk of death and relapse to three years, especially in patients with positive N1-N2 lymph nodes who underwent curative resection with D2 lymphadenectomy, but recurrence was more frequent in positive N3 lymph nodes and suggest employ new adjuvant protocols in this group of patients to decrease relapse rates. Favacho et al.^12^ analyzed 27 patients with staging T4b advanced gastric cancer who were only submitted to surgical treatment without adjuvant therapy and reported a 6-month survival of 63.27%.

In our opinion, for a better evaluation of the response of adjuvant chemoradiotherapy treatment, it is important to eliminate factors that may modify or create prognostic biases in the selected sample. Dosages of biomarkers such as HER2, microsatellite instability, Epstein-Barr virus, PD-L1 dosage and other markers may contribute to open new treatments and survival analysis, which associated with chemoradiotherapy, contribute to increased survival^29^.

Therefore, it should be emphasized that the treatment of gastric cancer is necessarily multidisciplinary^2^ ^,^ ^15^ and the survival analysis of the series suggests that the chemoradiation protocol proposed by Macdonald et al.^21^ may have benefits for patients with advanced disease undergoing surgery with curative intent and D2 lymphadenectomy. Additional studies with similar design are needed to define the real benefit of adjuvant chemoradiotherapy for the treatment of advanced gastric cancer.

## CONCLUSION

Adjuvant chemoradiotherapy presents survival gain for T4b patients undergoing surgical treatment with curative intent.
